# Bladder lipoma: Moroccan case report

**DOI:** 10.11604/pamj.2020.37.380.25672

**Published:** 2020-12-29

**Authors:** Omar Jendouzi, Younes Boukhlifi, Othmane El Houadfi, Mohamed Amine Essaoudi, Mohamed Alami, Ahmed Ameur

**Affiliations:** 1 Department of Urology, Military Teaching Hospital Rabat, Rabat, Morocco,; 2 Department of Pathology, Military Teaching Hospital Rabat, Rabat, Morocco

**Keywords:** Bladder, lipoma, benign neoplasm, case report

## Abstract

Lipomas are encapsulated benign tumors typically found in the integument, central nervous system or gastrointestinal tract and represent the most common benign mesenchymal neoplasm in adults. Bladder lipoma is a rare tumor that has been reported in a handful of cases in medical literature. A literature review from PubMed, MEDLINE, EMBASE and Cochrane databases of bladder lipoma yielded less than 20 cases. We report a case of a 69 year-old Moroccan male patient with hematuria as a chief symptom. The diagnosis of bladder lipoma was suspected by flexible fibroscopy and assessed by transurethral resection. Macroscopic and histological examination revealed a lipomatous tumour with no sign of malignancy. There was no recurrence after one year of follow-up. Although bladder lipomas are rare entities, they must be considered in the differential diagnosis of bladder tumor. However, we should always keep in mind that any bladder tumor is malignant until proven otherwise.

## Introduction

Conventional lipoma is the most common benign mesenchymal neoplasm in adults. However, bladder lipoma is a rare tumor, which has been reported in a handful of cases in medical literature. We presented an extremely rare case who was admitted to our department with hematuria and was incidentally diagnosed as bladder lipoma. Besides, a review of the literature on this subject is presented.

## Patient and observation

**Figure 1:**
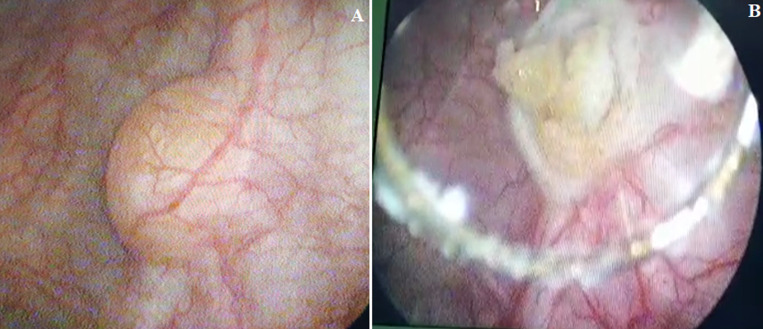
A) cystoscopy view of bladder lipoma approximately 5 mm in diameter; B) fatty appearance of the lesion during endoscopic resection

**Figure 2:**
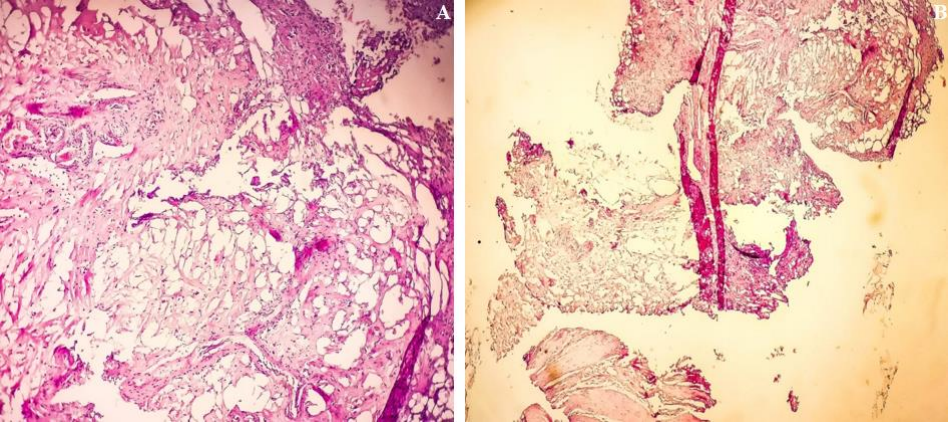
A) hemalun eosine of bladder showing a benign proliferation of mature adipose tissue, there are no mitotic figures in specimen suggesting, malignancy, magnification x 25; B) bladder musculature (detrusor) with abrasive coating on the surface and mature adipocyte proliferation: hemalun eosine, magnification x 10

A 69-year-old male patient was admitted to our hospital with chief symptom of gross haematuria (one episode). Urinary frequency and nocturia were other complaints. He had no comorbidity and was a non-smoker in his medical history. His physical examination, laboratory tests, and urine analysis were normal. His body mass index (BMI) was 24 kg/m^2^ (normal). No Transabdominal ultrasonography (US) or computed tomographic scan were performed. Therefore, we realised flexible cystoscopy that showed a smooth, yellow and benign-looking tumour, at the posterior wall of bladder measuring approximately 5 mm (Figure 1). There was no additional tumour. The lesion was seen as in the bladder, not out of bladder´s surface. The informed consent was obtained from patient. In the operation room, first spinal anaesthesia was performed and the genital area was sterilized in lithotomy position. The cystoscopy was indwelled (Figure 1). The tumour was suitable for resection in endoscopic route. Therefore, it was treated by transurethral resection. There was no residual tumour after endoscopic resection (Figure 2). The bleeding was controlled after resection and a three-way Foley catheter was inserted with soft continuous irrigation. The postoperative period went off without any incident to report. The patient was discharged on the first day of surgery. The clinical course was marked by the disappearance of the symptoms: no hematuria nor voiding symptoms during last 6 months were reported. The resection chips were analyzed by an experienced pathologist supervised by a senior and the diagnosis of lipoma was thus established. The description was in favor of a bladder wall showing connective and muscular tissue containing in one place a mature adipocyte proliferation (Figure 2 A and B). Liposarcoma was excluded from the outset because of the absence of atypical and immature lipoblasts.

## Discussion

Lipomas are encapsulated benign tumours typically found in the integument, central nervous system or gastrointestinal tract. The incidence of lipomas is particularly high in obesed patients suffering from diabetes mellitus or hypercholesterolemia but also in a family context [1]. The age category particularly affected by lipomas is between 40 and 60 years, but it can be seen in all age groups [1]. Most of bladder tumours (95%) originated from epithelium and often malignant. 5% are mesenchymal tumours. Among these, leiomyomas are the most common and account for 35% [2] while bladder lipoma are extremely rare [2]. A literature review from PubMed, MEDLINE, EMBASE and Cochrane databases of bladder lipoma yielded less than 20 cases [1]. The most common presentation is asymptomatic hematuria [3] and that was the main symptom of our case. The bleeding can be attributed to excoriations of the mucosa over the lipoma. Besides, bladder lipoma can manifest as urinary frequency, nocturia, urinary tract infection [4], or a retroperitoneal mass [5]; whatever the location, there are chromosomal rearrangements in chromosomes 12q, 6p and 13q in conventional lipomas [6], while pelvic lipomatosis seems to have a distinct genetic origin the rearrangements concern chromosomes 1 and 84 [5].

Lipomas were managed with cystoscopy and removed by biopsy or transurethral tumor resection. Ukita *et al*. [5], laparoscopically excised the exophytic bladder lipoma simulating an ovarian mature cystic teratoma. Most lipomas are superficial while deep or visceral lipomas are rare; they are and often discovered at an advanced stage of development and therefore tend to be larger than superficial lipomas. Yet, most of cases of bladder lipomas listed in international literature are small, measuring less than 2 cm and endophytic. Rarely Exophytic and may present as a retroperitoneal mass [5]. Lipomas may be located in every site of the bladder: in the posterior wall - as in our case - in the fundus or the dome [5]. Lipomas of the urinary bladder share common histopathological features with lipomas of the other tissue. Microscopically, lipomas are well-circumscribed, expansile neoplasms composed of mature adipose tissue without atypia [6]. Liposarcoma´s histological characters are nuclear atypia, multi-nucleated stromal cells, and occasional lipoblasts [7]. To the best of our knowledge, no bladder liposarcoma has been described.

Lipoma is a benign tumor and no case of malignant transformation has been reported in the literature [8]. All cases reviewed behaved as benign lesions and showed no recurrences [3], and this was the case with our patient after a follow up period of one year. Nevertheless, longterm follow-up may be necessary. Besides bladder, lipoma may be located in upper urinary tract (UUT): K Lmezguidi *et al*. reported a case of upper urinary lipoma in a 41-year-old man complaining of left flank pain. A computed tomographic urography revealed an irregular thickening of the left renal collecting system wall extending from the upper calices to the renal pelvis. The diagnosis of UUT was made and thereafter the patient underwent a nephroureterectomy with bladder cuff excision, macroscopic and histological examination revealed however a lipomatous tumour with no sign of malignancy [9]. Bladder lipoma should be differentiated from pelvic lipomatosis which was first reported in the literature by Arikan *et al*. [10]. Multiple lipomatous lesions arise from peri-rectal and peri-vesical adipose tissue and may cause compression of the pelvic viscera, including the urethra and, in some cases the ureters, resulting in uremia. Computed tomography and MRI are useful for diagnosis [8]. The key-point is to consider the possibility of a well-differentiated liposarcoma and avoid any rupture of the capsule while the excision of the mass.

## Conclusion

Bladder lipoma is a rare finding within bladder and have been described in only handful cases of literature. The clinical presentation is non-specific. Cystoscopy is essential after an episode of gross hematuria to evaluate the bladder for urothelial tumours in order to accurate the diagnosis and offer adequate treatment.
